# Pancreatic Fat Accumulation Impacts Postoperative Survival in Patients With Pancreatic Ductal Adenocarcinoma

**DOI:** 10.1002/wjs.12576

**Published:** 2025-04-03

**Authors:** Yasunari Fukuda, Chikato Koga, Soichiro Minami, Satoshi Ishikawa, Atsushi Gakuhara, Shuichi Fukuda, Naotsugu Haraguchi, Jinichi Hida, Tomoko Wakasa, Yutaka Kimura

**Affiliations:** ^1^ Department of Gastroenterological Surgery Kindai University Nara Hospital Nara Japan; ^2^ Department of Pathology Kindai University Nara Hospital Nara Japan

**Keywords:** fat accumulation, fatty pancreas, pancreatic cancer, prognosis, survival

## Abstract

**Background:**

Pancreatic fat accumulation, that is, fatty pancreas (FP), has gained attention because it contributes to pancreatic carcinogenesis. However, the impact of FP on the survival of patients with pancreatic cancer has not yet been elucidated.

**Methods:**

Overall, 87 consecutive patients who were pathologically diagnosed with pancreatic ductal adenocarcinoma and underwent potentially curative pancreatectomy were eligible for analysis. Histological pancreatic fat fraction (HPFF) was evaluated using hematoxylin & eosin‐stained slides of tumor and non‐tumor sections of the resected specimen, and quantified using imaging analysis software. The optimal HPFF value threshold for FP presence was determined using receiver operating characteristics curve analysis. The prognostic significance of FP was identified by a Cox proportional hazard model adjusted for the established prognostic covariates.

**Results:**

Fat accumulation within the invasive tumor front was scarce, with the median value for HPFF being 10.1% in the non‐tumor portion (range: 2.2%–45.8%). Patients with FP (HPFF value ≥ 11.3%) in the non‐tumor portion had significantly inferior overall survival (OS) and recurrence‐free survival (RFS) than those without FP (HPFF value < 11.3%) (log‐rank test: *p* = 0.012 and *p* = 0.00060, respectively). In the multivariate analyses, the presence of FP emerged as an independent inferior prognostic indicator (OS: hazard ratio [HR] 2.32, *p* = 0.0015; PFS: HR 2.33, *p* = 0.00080), together with lymph node metastases, presence of lymphatic involvement, and absence of adjuvant chemotherapy.

**Conclusion:**

The present study indicates a possible prognostic role for pancreatic fat accumulation in patients with pancreatic adenocarcinoma.

## Graphical abstract

Fatty pancreas was pathologically assessed and quatificated using tumor and non‐tumor portions of resected specimens in patients with pancreatic cancer. We found that the presence of fatty pancreas evaluated in non‐tumor portions was an independent inferior prognostic indicator for overall survival and recurrence free survival.

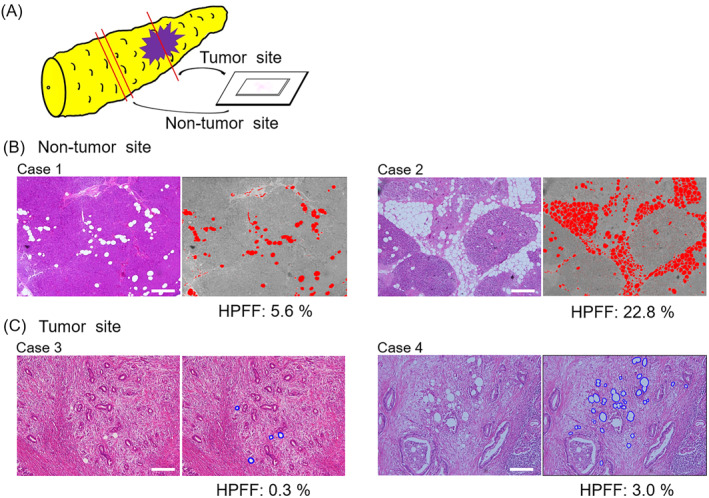

## 1 Introduction

The increasing prevalence of excessive weight and obesity has become a major global health concern over the last few decades [1]. Excessive weight and obesity can occasionally contribute to ectopic fat accumulation in parenchymatous organs, including skeletal muscle, liver, heart, and pancreas [2]. Ectopic fat accumulation is an emerging clinical entity owing to the associated hallmark metabolic abnormalities. In particular, pancreatic fat accumulation, that is, fatty pancreas (FP), is directly linked with the pathogenesis of diabetes mellitus, being the consequence of disturbed β‐cell function and insulin resistance [3, 4]. Furthermore, fat accumulation in the liver and pancreas is considered to be a metabolic factor predisposing individuals to cancer, although the specific pathophysiological mechanisms remain incompletely understood [5, 6, 7, 8, 9].

Prior studies, including those from our research group, have highlighted the impact of FP on the occurrence of pancreatic cancer, assessed by pathological and radiographical approaches [6, 7]. Our recent study suggested that computed tomography (CT) number and proton density fat fraction using 3‐T magnetic resonance imaging can quantitatively evaluate histological fat accumulation, and these radiographic modalities may be useful for the stratification of individuals at high‐risk of pancreatic cancer according to their FP diagnosis [7, 10]. In addition, a recent meta‐analysis reported that the prevalence of FP was six‐fold higher among patients with pancreatic cancer compared to healthy individuals [11]. However, the clinical impact of FP on patients with pancreatic cancer remains unclear.

Adipocytes can promote carcinogenesis and cancer progression. Intricate crosstalk between adipocytes, cancer cells, and immune cells, mediated via various bioactive substances that include those secreted by adipocytes, influences the proliferative and metastatic potential of cancer cells, particularly in an adipocyte‐rich environment [12, 13, 14, 15]. Indeed, several adipokines have been reported to boost pancreatic cancer progression and affect patient prognosis [16, 17, 18], indicating the distinctive contribution of FP to pancreatic cancer pathophysiology. In a study using a mouse model with a mutated Kras G12D, fed with a high‐fat diet developed significantly larger pancreatic tumors and demonstrated an increased rate of distant metastasis compared to those fed with a standard diet [17]. Prior studies have demonstrated that pancreatic fat, assessed using histological examination and CT imaging, was significantly more abundant in patients with pancreatic cancer with nodal metastases compared to those without them, supporting the hypothesis that FP promotes cancer progression [19, 20].

Therefore, we hypothesized that FP may impact the survival of patients with pancreatic cancer. In this study, we investigated whether pancreatic fat accumulation, examined at the tumor and non‐tumor portions of resected specimens, was associated with postoperative survival in patients with pancreatic cancer after radical pancreatectomy.

## 2 Patients and Methods

### 2.1 Study Participants

We consecutively screened 95 patients who were pathologically diagnosed with conventional pancreatic invasive ductal adenocarcinoma (PDAC) and underwent pancreatectomy between April 2000 and May 2022 at Kindai University Nara Hospital. Variants of PDAC and intraductal papillary mucinous carcinomas were not included owing to different prognostic implications. Among the original patient cohort, eight patients were excluded from the final analysis: one patient with concurrent distal bile duct carcinoma; one who underwent R2 resection; two with Clavien‐Dindo Grade V complications; and three for whom histological FP could not be assessed. Accordingly, the remaining 87 patients were analyzed in the present study. The study protocol was reviewed and approved by the Ethics Committee of Kindai University Nara Hospital (approval number:24‐4).

### 2.2 Histological Assessment of Pancreatic Fat Accumulation in the Resected Specimens

**FIGURE 1:**
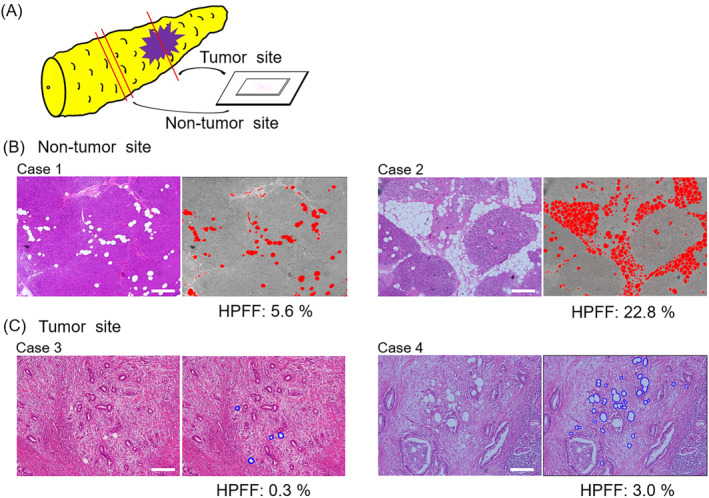
Histological assessment of pancreatic fat accumulation (A) Illustration of a resected specimen of the pancreas. H&E‐stained slides were generated from tumor and non‐tumor portions (one slide from the tumor portion and two from the non‐tumor portion). (B) Illustrative H&E‐stained images of the non‐tumor site from two different patients with PDAC. In each case, intralobular and interlobular lipid droplets were automatically recognized with red‐colored area using Image J. HPFF (%) was calculated as the ratio of the total red‐colored area to the entire area. Intralobular lipid droplets were observed in Case 1 (HPFF value: 5.6%), and intra‐ and interlobular lipid droplets were observed in Case 2 (HPFF value: 22.8%). Scale bar = 500 μm. (C) Illustrative H&E‐stained images of the tumor site from two different patients with PDAC. In each case, lipid droplets within the invasive front were manually circled in blue, and the circled area was measured using Image J. HPFF (%) was calculated as the ratio of the total area circled in blue to the entire area. HPFF values were 0.3% and 3.0% in Cases 3 and 4, respectively. Scale bar = 500 μm. HPFF, histological pancreatic fat fraction; H&E, hematoxylin & eosin; PDAC, pancreatic ductal adenocarcinoma.

The histological pancreatic fat fraction (HPFF) in the resected specimens was assessed using 3 μm thick hematoxylin & eosin (H&E) stained slides of tumor and non‐tumor portion of surgical specimens. Specifically, the HPFF of the tumor portion was evaluated at the invasive tumor front and that of the non‐tumor portion at the proximal side of the pancreas, away from the tumor to avoid contamination with tumor‐induced pathological alterations (Figure 1A). One slide of the tumor portion and two slides of the non‐tumor portion were obtained from each patient by one of the authors (Y.F.), and the quantitative analysis was performed with the researchers blinded to the clinicopathological information from each participant. The quality of each slide was determined by the expert pathologist of our institute (T.W.).

### 2.3 Quantification of Histological Pancreatic Fat Fraction

Two different areas were randomly scanned from each slide using light microscopy at x 40 magnification (BX50 OLYMPUS, Tokyo, Japan). The intra‐ and interlobular fat droplets at the non‐tumor portion were automatically recognized using an image analysis software (ImageJ version 1.51; https://imagej.nih.gov/) (Figure 1B). Fat can be found in two different areas within the tumor tissue: extra‐pancreatic fat adjacent to the tumor area (Figure S1A) and fat within the invasive tumor front (Figure S1B). We focused exclusively on the latter in this study. Lipid droplets within the tumor area were manually identified by Y.F. and T.W., since the blank areas within the ductal structure of the tumor were not automatically discriminated from true lipid droplets by the image analysis software (Figure 1C). HPFF values (%) at the tumor and non‐tumor portions were calculated as the ratio of the lipid droplet area to the entire area. Since one slide of the tumor portion and two of the non‐tumor portion were selected, the averages of two and four data values of HPFF were calculated as the HPFF value of the tumor and non‐tumor portions, respectively.

### 2.4 Covariates for Prognostic Analysis

The present study included age, sex, preoperative serum CA19‐9 value, curability (R status), presence of neoadjuvant and adjuvant chemotherapy, postoperative major complications, postoperative pancreatic fistula (POPF), pathological T stage, lymph node positivity, lymphatic invasion, vessel invasion, neural invasion, tumor differentiation, and presence of FP as covariates for the identification of prognostic indicators. Neoadjuvant chemotherapy with either single‐agent gemcitabine, gemcitabine combined with tegafur‐gimeracil‐oteracil Potassium (S‐1), nab‐paclitaxel combined with gemcitabine, or FOLFIRINOX was selected, according to the criteria of the physician managing each patient. Adjuvant chemotherapy with either gemcitabine or S‐1 was administered whenever indicated. Patients who received at least two cycles of these regimens were classified as having received neoadjuvant or adjuvant chemotherapy. Postoperative complications classified as Clavien‐Dindo grade IIIa or higher were considered major complications. In terms of POPF, we focused on clinically relevant POPF (grade B and C) based on the International Study Group on Pancreatic Fistula classification [21].

### 2.5 Statistical Analysis

All **s**tatistical analyses were performed using JMP software (SAS Institute, Cary, NC, USA). Continuous variables are reported as median (range). Categorical variables were compared using the chi‐squared test, and continuous variables using Student's *t*‐test or the Mann–Whitney *U* test, as appropriate. The probabilities of postoperative survival were estimated using the Kaplan–Meier method and compared using the log‐rank test. Univariate and multivariate analyses were conducted using a Cox proportional hazard model to determine the prognostic significance of the covariates. Prognostic covariates showing significance in the univariate analyses were included in the multivariate analyses. The optimal cutoff value of the HPFF for the presence of FP was determined by receiver operating characteristics (ROC) curve analyses based on 2‐year survival as the outcome. A two‐sided *p*‐value was used for all the analyses, and statistical significance was set at *p* < 0.05.

## 3 Results

### 3.1 Clinicopathological Demographics of Study Participants

The preoperative baseline demographic data are shown in Table 1. The median age of our cohort was 72 years (range: 47–85), with approximately equal proportions of men and women. Neoadjuvant and adjuvant chemotherapies were administered to 12 (13.8%) and 57 (65.5%) patients, respectively. Among those who received adjuvant chemotherapy, 47 patients (82.5%) were treated with S‐1. The most frequent surgical procedure in this cohort was subtotal stomach‐preserving pancreatoduodenectomy (SSPPD; 74.7%), and R0 resection was achieved in 62 patients (71.3%). The frequency of major complications and POPF was 26.4% and 13.8%, respectively. The median HPFF value for the non‐tumor portion was 10.1% (range: 2.2%–45.5%). In contrast, lipid droplets were scarce in the invasive front of the tumor (median HPFF value: 0.0%).

**TABLE 1:** Clinicopathological characteristics of the study participants.

	*n* = 87	
	Median/number	Range/%
Age, years	72	47–85
Sex		
Male	45	51.7
Female	42	48.3
BMI, kg/m^2^	22.1	15.0–29.9
Serum CA19–9, U/mL	85.1	0.8–6980.6
Serum total cholesterol, mg/dL	183	83–312
Serum triglycerides, mg/dL	111	37–299
Neoadjuvant chemotherapy		
Presence	12	13.8
Absence	75	86.2
Adjuvant chemotherapy		
Presence	57	65.5
Absence	30	34.5
Type of surgery		
SSPPD	65	74.7
DP	21	24.1
TP	1	1.1
Major complication	23	26.4
Postoperative pancreatic fistula	12	13.8
R status		
R0	62	71.3
R1	25	28.7
T stage		
T1	5	5.7
T2	7	8.0
T3	74	85.1
T4	1	1.1
Lymph node positivity		
N0	39	44.8
N1	48	55.2
Lymphatic invasion		
ly0	13	14.9
ly1–3	74	85.1
Vessel invasion		
v0	44	50.6
v1–3	43	49.4
Neural invasion		
ne0	17	19.5
ne1–3	70	80.5
Tumor differentiation		
Well/moderate	80	92.0
Poor	7	8.0
HPFF value at non‐tumor site, %	10.1	2.2–45.8
HPFF value at tumor site, %	0.0	0.0–2.7

Abbreviations: BMI, body mass index; DP, distal pancreatectomy; HPFF, histological pancreatic fat fraction; SSPPD, subtotal stomach‐preserving pancreaticoduodenectomy; TP, total pancreatectomy.

### 3.2 Optimal HPFF Cutoff Value for the Definition of FP

Fat accumulation at the tumor portion was too scarce to evaluate its association with patient survival; therefore, only fat accumulation at the non‐tumor portion was subjected to the FP assessment and survival analyses. ROC analyses were performed to establish the optimal threshold of the HPFF value in defining FP, and the Youden index was highest with an HPFF value of 11.3% (area under the curve [AUC], 0.69; sensitivity, 0.79; specificity, 0.63; Figure S2).

### 3.3 Relationship Between Presence of FP and Patient Characteristics

Overall, 38 patients with an HPFF value of ≥ 11.3% at the non‐tumor portion were categorized as having FP (FP group), with the remaining 49 classified as without FP (HPFF value of < 11.3%; non‐FP group). Table 2 shows the differences in clinicopathological features between the two groups. Age and sex ratio was similar between the two groups (*p* = 0.31 and 0.31, respectively). FP has been reported to be associated with body composition [7] and lipid status [22]; however, no significant differences in BMI were observed in the present cohort (*p* = 0.18). Neither serum levels of total cholesterol nor those of triglycerides were associated with FP. The FP group showed a trend toward increased occurrence of POPF (*p* = 0.084). Although this is likely to be linked to the administration of adjuvant chemotherapies, delayed start of adjuvant chemotherapies, and its completion rate, which may influence postoperative survival [23], the occurrence of POPF did not affect the administration rate of adjuvant chemotherapies [8 out of 12 patients in the POPF group: 66.6% *vs.* 49 out of 75 patients in the non‐POPF group, 65.3%; *p* = 0.93] or the initiation of adjuvant chemotherapies (initiated with a median delay of 53.5 and 50.5 days in patients with and without POPF, respectively; *p* = 0.42) as well as its completion rate (4 out of 8 patients in the POPF group, 50.5% vs. 35 out of 49 patients in the non‐POPF group, 71.4%; *p* = 0.23). Patients in the FP group possessed advanced tumor features, although no feature reached statistical significance.

**TABLE 2:** Clinicopathological characteristics of patients according to the presence of FP.

	*n* = 87		*p* value
	Non‐FP (*n* = 49)	FP (*n* = 38)	
Age, years	71 (47–84)	73 (53–85)	0.31
Sex			
Male/female	23/26	22/16	0.31
BMI, kg/m^2^	21.7 (15.0–26.6)	22.2 (16.0–29.9)	0.18
Serum CA19–9, U/mL	65.1 (0.8–6908.6)	96.4 (2.0–3608.0)	0.13
Serum total cholesterol, mg/dL	180 (105–299)	187 (83–312)	0.70
Serum triglycerides, mg/dL	112 (40–290)	109 (37–299)	0.69
Neoadjuvant chemotherapy			
Presence/absence	5/44	7/31	0.27
Adjuvant chemotherapy			
Presence/absence	33/16	24/14	0.68
Major complication			
Presence/absence	12/37	11/27	0.64
Postoperative pancreatic fistula			
Presence/absence	4/45	8/30	0.084
T stage			
T1, 2/T3, 4	9/40	3/35	0.16
LN positivity			
N0/N1	24/25	15/23	0.38
Lymphatic invasion			
ly0/ly1–3	9/40	4/34	0.30
Vessel invasion			
v0/v1–3	29/20	15/23	0.067
Neural invasion			
ne0/ne1–3	10/39	7/31	0.82
Tumor differentiation			
Well, moderate/poor	46/3	34/4	0.46

Abbreviations: BMI, body mass index; FP, fatty pancreas; LN, lymph node.

### 3.4 Postoperative Survival Significance of FP in Patients With PDAC

**FIGURE 2:**
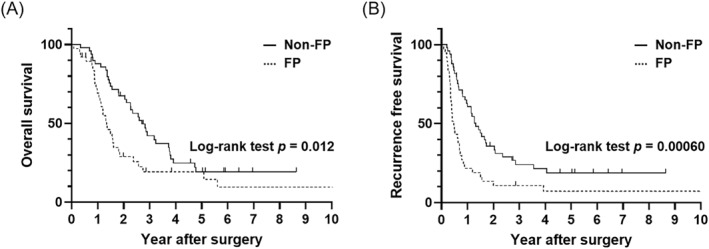
Kaplan–Meier survival curve stratified by FP presence (A) Patients with FP had significantly worse postoperative OS than those without FP (MST 1.34 vs. 2.71 years; log‐rank test *p* = 0.012). (B) Patients with FP had significantly worse postoperative RFS than those without FP (MST 0.48 vs. 1.32 years; log‐rank test *p* = 0.00060). FP, fatty pancreas; MST, median survival time; OS, overall survival; RFS, recurrence‐free survival.

The median survival time (MST) of the entire cohort was 1.85 years. Patients in the FP group had significantly worse overall survival (OS) than those in the non‐FP group (MST: 1.34 vs. 2.71 years, log‐rank test, *p* = 0.012), as shown in Figure 2A. Similarly, recurrence‐free survival (RFS) was inferior in patients with FP compared to those without FP (MST: 0.48 vs. 1.32 years, log‐rank test, *p* = 0.00060; Figure 2B). Subsequently, univariate and multivariate analyses using a Cox proportional hazard model were conducted to determine the prognostic significance of FP in patients with PDAC. The cutoff value for serum CA19‐9 (230.0 U/mL) was based on our previous study [24]. In the univariate analyses, the serum CA19‐9 value, pathological T stage, LN metastases, lymphatic invasion, adjuvant chemotherapy, and FP were significantly associated with postoperative OS. In addition, R status was significant for RFS. In the multivariate analyses, the presence of FP was identified as an independent unfavorable prognostic indicator for OS and RFS (OS: hazard ratio [HR] 2.32, 95% confidence interval [CI] 1.38–3.91, *p* = 0.0015; and PFS: HR 2.33, 95% CI 1.42–3.81, *p* = 0.00080), together with the presence of LN metastases, lymphatic invasion, and the absence of adjuvant chemotherapy (Tables 3 and 4).

**TABLE 3:** Prognostic indicators associated with postoperative overall survival in patients with PDAC.

Variable		Univariate			Multivariate		
		HR	95% CI	*p* value	HR	95% CI	*p* value
Age	≥ 75 versus < 75 years	1.11	0.68–1.83	0.67			
Sex	Male versus female	1.60	0.98–2.64	0.75			
Serum CA19‐9	≥ 230.0 versus < 230.0 U/ml	1.73	1.03–2.90	0.038	1.15	0.67–1.99	0.61
R status	R1 versus R0	1.46	0.87–2.45	0.15			
Major complication	Presence versus absence	0.84	0.47–1.49	0.55			
Postoperative pancreatic fistula	Presence versus absence	0.57	0.24–1.32	0.19			
T stage	T3, 4 versus T1, 2	2.49	1.07–5.79	0.034	1.70	0.71–4.07	0.23
LN positivity	N1 versus N0	2.50	1.49–4.18	0.00050	2.50	1.42–4.38	0.0014
Lymphatic invasion	ly1–3 versus ly0	3.33	1.43–7.77	0.0053	3.07	1.21–7.81	0.019
Vessel invasion	v1–3 versus v0	1.58	0.97–2.58	0.065			
Neural invasion	ne1–3 versus ne0	1.70	0.89–3.25	0.11			
Tumor differentiation	Poor versus well, moderate	1.55	0.69–3.16	0.27			
Neoadjuvant chemotherapy	Presence versus absence	0.65	0.28–1.52	0.32			
Adjuvant chemotherapy	Presence versus absence	0.41	0.25–0.68	0.00060	0.24	0.13–0.42	< 0.0001
FP	Presence versus absence	1.84	1.15–3.09	0.011	2.32	1.38–3.91	0.0015

Abbreviations: CI, confidence interval; FP, fatty pancreas; HR, hazard ratio; LN, lymph node; PDAC, pancreatic ductal adenocarcinoma.

**TABLE 4:** Prognostic indicators associated with postoperative recurrence free survival in patients with PDAC.

Variable		Univariate			Multivariate		
		HR	95% CI	*p* value	HR	95% CI	*p* value
Age	≥ 75 versus < 75 years	0.98	0.60–1.59	0.93			
Sex	Male versus female	1.29	0.80–2.07	0.30			
Serum CA19‐9	≥ 230.0 versus < 230.0 U/ml	1.96	1.18–3.27	0.0098	1.07	0.61–1.85	0.82
R status	R1 versus ≥ R0	1.80	1.08–2.99	0.024	1.49	0.85–2.61	0.16
Major complication	Presence versus absence	0.78	0.44–1.38	0.39			
Postoperative pancreatic fistula	Presence versus absence	0.58	0.25–1.34	0.20			
T stage	T3, 4 versus < T1, 2	2.74	1.18–6.36	0.019	1.49	0.61–3.62	0.38
LN positivity	N1 versus N0	2.71	1.46–4.48	0.00010	2.59	1.46–4.58	0.0011
Lymphatic invasion	ly1–3 versus ly0	4.11	1.64–10.26	0.0025	2.86	1.07–7.62	0.036
Vessel invasion	v1–3 versus v0	1.44	0.90–2.32	0.13			
Neural invasion	ne1–3 versus ne0	1.69	0.90–3.16	0.10			
Tumor differentiation	Poor versus well, moderate	2.00	0.91–4.42	0.084			
Neoadjuvant chemotherapy	Presence versus absence	0.70	0.32–1.53	0.37			
Adjuvant chemotherapy	Presence versus absence	0.42	0.26–0.69	0.00060	0.27	0.16–0.47	< 0.0001
FP	Presence versus absence	2.27	1.40–3.66	0.00080	2.33	1.42–3.81	0.00080

Abbreviations: CI, confidence interval; FP, fatty pancreas; HR, hazard ratio; LN, lymph node; PDAC, pancreatic ductal adenocarcinoma.

## 4 Discussion

Pancreatic cancer is one of the deadliest types of cancer, and has shown rapidly increasing incidence in the last decade, which may be associated with the global obesity epidemic. Therefore, a rising demand for research on the development and progression of pancreatic cancer from a lipid perspective exists to advance early detection, prognostic prediction, and effective treatments for this cancer. To the best of our knowledge, this is the first study to elucidate the prognostic role of FP on postoperative survival in patients with PDAC. Mathur et al. previously demonstrated that the abundance of adipocytes in the pancreas was higher in patients with lymph node‐positive PDAC than in those with node‐negative PDAC after matching for several clinical characteristics, and patient demise occurred earlier in node‐positive cases; however, this study did not report a direct causal relationship between FP and the survival of patients with PDAC [19, 20].

Infiltration of adipocytes has been identified in the intra/interlobular spaces of the pancreatic acinar parenchyma [25, 26]. Interlobular adipocytes are predominantly adjacent to arterioles. Furthermore, intra‐acinar cytoplasmic vacuoles have also been detected in both humans and mice [27]. In the present study, we only measured intra/interlobular lipid droplets because we were unable to perform the quantification of intra‐acinar vacuoles. Intriguingly, different roles for intra‐ and interlobular fat infiltration in the progression of PDAC and pancreatic intraepithelial neoplasia (PanIN) lesions have been proposed [8, 28]. Intralobular and interlobular adipocytes conferred different lipidomic profiles with different roles in the oncogenic process: intralobular adipocytes contributed to acinar modifications, inducing oxidative stress and activation of inflammatory pathways, and predisposing to the development of PanIN lesions, whereas interlobular adipocytes were responsible for the progression of PDAC [28]. Intra and interlobular lipid droplets were not easy to separate for quantitative analyses in our series; nevertheless, future studies on these different types of adipocytes, including intra‐acinar adipocytes, may inform new approaches for PDAC detection, prognostication, and treatment.

Our study evaluated the fat present in the vicinity of the tumor, since adipocytes that surround tumor cells, (“cancer‐associated adipocytes”) can develop specific characteristics that support tumor growth and have been found to be associated with patient prognosis [29, 30, 31]. The infiltrating pattern of these adipocytes varies according to individual cases as well as cancer type [32]. Extra‐pancreatic fat adjacent to tumor cells due to the extra‐pancreatic invasion of cancer cells is a possible candidate for analysis. However, we excluded this fat from the quantitative analyses because this amount is largely dependent on the extent of resection. On the other hand, scarce infiltration of adipocytes was observed within the invasive tumor front, likely because pancreatic cancer is a scirrhous type of carcinoma, and intra‐tumoral fat is easily replaced or pushed aside by the abundant fibrous stroma.

Several mechanisms may explain the role of PF on the survival of patients with PDAC. Emerging evidence has suggested that several adipokines released from adipocytes can serve as energy sources for cancer cell growth [12, 13, 14, 15, 16, 17, 18]. Leptin, a key adipokine that shows elevated levels in the blood of patients with FP [33], has been suggested to induce upstream activation of the notch pathway, which is conducive to the increase in tumorigenesis and stimulate the growth of pancreatic cancer stem cell populations [18]. Furthermore, adipocytes support the establishment of an immunosuppressive milieu by promoting the accumulation of M2‐polarized macrophages, regulatory T cells, and myeloid‐derived suppressor cells [14, 34]. Considering that FP may result in local release of abundant adipokines into the tumor microenvironment, the presence of FP may significantly modulate the metabolic characteristics of tumor cells and the tumor immune microenvironment in favor of cancer progression and metastasis, affecting patient survival. However, it is also possible that FP may be just a surrogate of systemic fat deposition. Further research with a particular emphasis on FP biology is crucial for determining the significance of FP in pancreatic cancer.

Some limitations of the study should be considered. First, the current study did not describe the clinical characteristics of FP capable of affecting postoperative survival. Although patients with FP had a tendency to develop POPF, this complication did not directly influence the administration of adjuvant chemotherapies. Second, our study only included Japanese patients, and none of them had been diagnosed with obesity (defined as having a BMI of 30 kg/m^2^ or higher). Caution should therefore be taken when generalizing our findings to European or North American patient cohorts, since the prevalence of overweight and obese individuals is markedly different in those areas of the globe. Third, this was a single‐center, retrospective, observational study, and the sample size was small. No significant correlations between FP and factors related to body fat were identified, in contrast to our previous research [7]. This may be related to the small sample size in the present study. In addition, advances in surgical techniques and chemotherapy during the long timeframe of the study (from 2000 to 2022) may have also affected the significance of FP on postoperative survival. Large prospective cohort studies are critical to gather additional evidence on the impact of FP on postoperative survival in patients with pancreatic cancer.

In conclusion, the results from the present study suggest that pancreatic fat accumulation influences postoperative survival in patients with PDAC. Pancreatic fat accumulation is considered to be a potentially reversible metabolic state [35]. Therefore, improved lifestyle measures and sustained conservative medical management should be encouraged in patients with FP to minimize the risk of developing pancreatic cancer and control cancer progression once it is present.

## Author Contributions

**Yasunari Fukuda:** conceptualization, data curation, formal analysis, investigation, writing – original draft, writing – review and editing. **Chikato Koga:** data curation, formal analysis, supervision. **Soichiro Minami:** data curation, formal analysis, investigation. **Satoshi Ishikawa:** data curation, formal analysis, investigation. **Atsushi Gakuhara:** data curation, formal analysis, investigation. **Shuichi Fukuda:** data curation, formal analysis, investigation. **Naotsugu Haraguchi:** data curation, formal analysis, investigation, supervision. **Jinichi Hida:** data curation, formal analysis, investigation. **Tomoko Wakasa:** data curation, methodology, supervision. **Yutaka Kimura:** investigation, supervision.

## Conflicts of Interest

The authors declare no conflicts of interest.

## Supporting information

Figure S1

Figure S2

Supporting Information S1

## Data Availability

Data available on request from the authors.
