# Supplementary figures and images for: Pancreatic Fat Accumulation Impacts Postoperative Survival in Patients With Pancreatic Ductal Adenocarcinoma
Source: World J Surg. 2025 Apr 3;49(5):1327–35. doi: 10.1002/wjs.12576 (PMC12058435; doi:10.1002/wjs.12576)

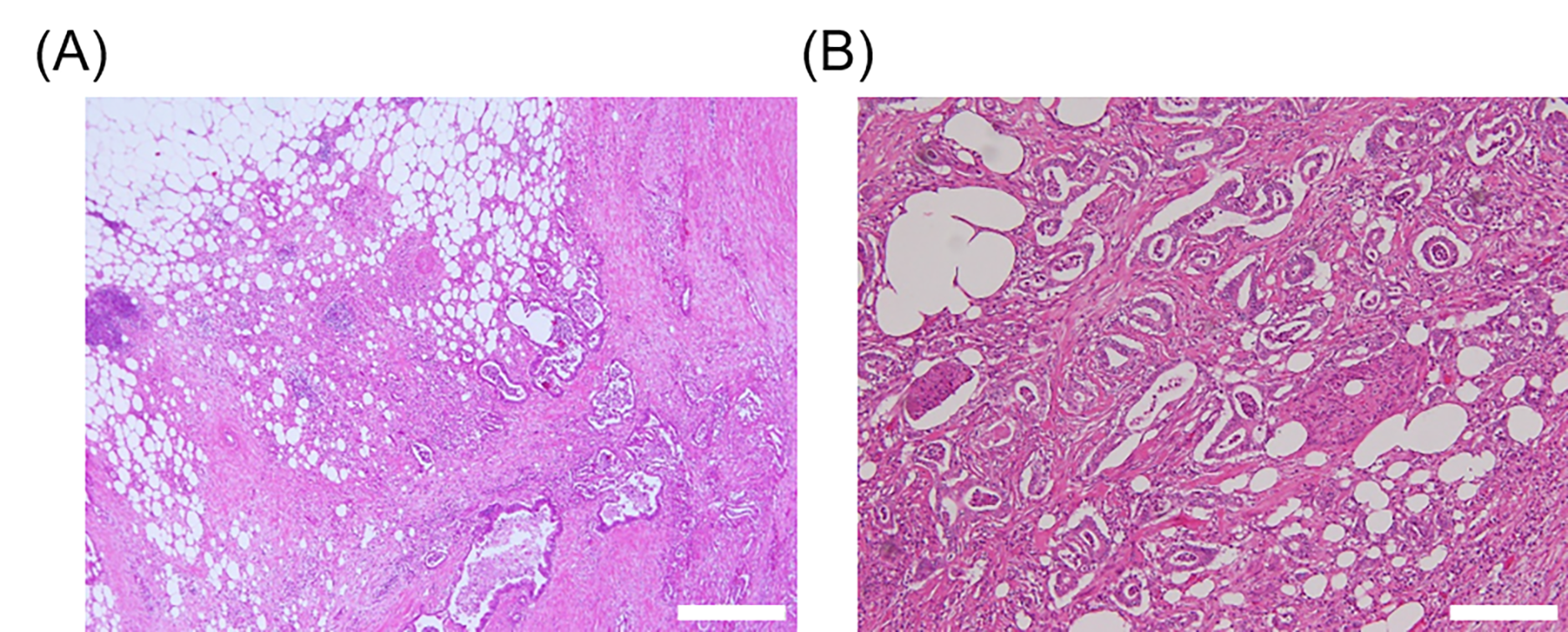

Supplement: Supplementary file 1 — Figure S1 [file WJS-49-1327-s003.tif]

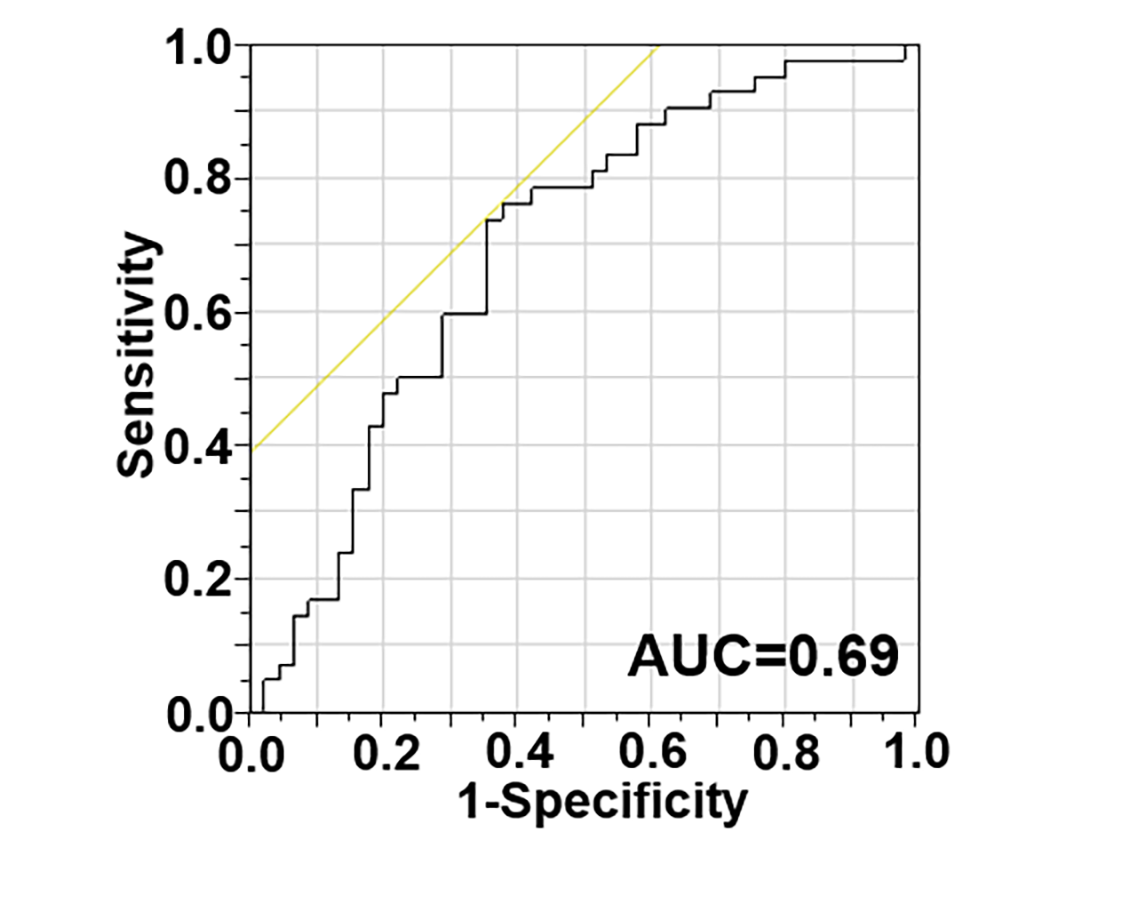

Supplement: Supplementary file 2 — Figure S2 [file WJS-49-1327-s001.tif]
