# Supplementary material for: Pancreatic Fat Accumulation Impacts Postoperative Survival in Patients With Pancreatic Ductal Adenocarcinoma
Source: World J Surg. 2025 Apr 3;49(5):1327–35. doi: 10.1002/wjs.12576 (PMC12058435; doi:10.1002/wjs.12576)
Supplement: Supplementary file 3 — Supporting Information S1 [file WJS-49-1327-s002.docx]

**Supplementary figure legends**

**Supplementary Fig. 1 Two different patterns of peritumoral fat accumulation**

a) Illustrative H&E-stained image of extra-pancreatic fat invaded by tumor cells.

Scale bar = 500 μm.

b) Illustrative H&E-stained image of fat within the front of tumor invasion.

Scale bar = 500 μm.

*H&E:* hematoxylin & eosin

**Supplementary Fig. 2 ROC curve for the definition of FP**

The optimal cutoff value of the HPFF for the definition of FP presence was 11.4%, which yielded a sensitivity of 0.79 and specificity of 0.63, with an AUC of 0.69.

*AUC:* area under the curve, *HPFF:* histological pancreatic fat fraction, *FP:* fatty pancreas, *ROC:* receiver operating characteristics
